# Membrane Distillation Hybrid Peroxydisulfate Activation toward Mitigating the Membrane Wetting by Sodium Dodecyl Sulfate

**DOI:** 10.3390/membranes12020164

**Published:** 2022-01-29

**Authors:** Minyuan Han, Ruixue Zhao, Jianchao Shi, Xiaobo Li, Daoling He, Lang Liu, Le Han

**Affiliations:** 1 Key Laboratory of the Three Gorges Reservoir Region’s Eco-Environment, Ministry of Education, College of Environment and Ecology, Chongqing University, Chongqing 400045, China; MinhuanHan@163.com (M.H.); zhaorx2022@163.com (R.Z.); 2 School of Civil Engineering, Yantai University, Yantai 264005, China; shijianchao@ytu.edu.cn; 3 Animal Husbandry Service of Chongqing, Chongqing 401121, China; lixb616121@163.com (X.L.); hdl010308@163.com (D.H.); 4 State Key Laboratory of Pollution Control and Resource Reuse, School of the Environment, Nanjing University, Nanjing 210023, China; l.liu@cqu.edu.cn

**Keywords:** membrane distillation, peroxydisulfate activation, surfactant, (anti-)wetting, electrochemical impedance spectroscopy

## Abstract

The fouling/wetting of hydrophobic membrane caused by organic substances with low-surface energy substantially limits the development of the membrane distillation (MD) process. The sulfate radical ($SO_{4}^{\;\cdot -}$)-based advanced oxidation process (AOP) has been a promising technology to degrade organics in wastewater treatment, and peroxydisulfate (PDS) could be efficiently activated by heat. Thus, a hybrid process of MD-AOP via PDS activated by a hot feed was hypothesized to mitigate membrane fouling/wetting. Experiments dealing with sodium dodecyl sulfate (SDS) containing a salty solution via two commercial membranes (PVDF and PTFE) were performed, and varying membrane wetting extents in the coupling process were discussed at different PDS concentrations and feed temperatures. Our results demonstrated permeate flux decline and a rise in conductivity due to membrane wetting by SDS, which was efficiently alleviated in the hybrid process rather than the standalone MD process. Moreover, such a mitigation was enhanced by a higher PDS concentration up to 5 mM and higher feed temperature. In addition, qualitative characterization on membrane coupons wetted by SDS was successfully performed using electrochemical impedance spectroscopy (EIS). The EIS results implied both types of hydrophobic membranes were protected from losing their hydrophobicity in the presence of PDS activation, agreeing with our initial hypothesis. This work could provide insight into future fouling/wetting control strategies for hydrophobic membranes and facilitate the development of an MD process.

## 1. Introduction

Membrane distillation (MD) is a thermal desalination process that uses microporous hydrophobic membrane to separate the hot, salty feed and cold distillate. Under the vapor pressure difference induced by the temperature gradient, the water vapor is transported across the membrane and condenses into distillate [1,2,3]. Compared to other pressure-driven, membrane-based desalination technologies (e.g., reverse osmosis), the advantages of MD include the utilization of recyclable heat or solar energy, low sensitivity to feed salinity, affordable operating conditions such as low pressure and moderate temperature [4]. Nevertheless, the membrane fouling/wetting phenomenon has been a great obstacle for MD, particularly in dealing with feed solution containing amphiphilic molecules (e.g., surfactants) even at low concentrations [3,5,6,7]. Specifically, surfactants are widely used in household goods, detergents, and many other aspects in modern industrial activities [8]. They could readily adhere to the membrane surfaces in MD via hydrophobic and/or electrostatic interactions, gradually sacrificing the pore hydrophobicity during the propagation of the liquid–air interface under the liquid entry scenario [9,10,11]. Despite the development of bespoke MD membranes with special wettability being imperative to overcome these issues, solution polishing to reduce its wetting propensity via adequate pretreatments is also a reasonable strategy [12,13].

Advanced oxidation processes (AOPs) have been reported to effectively degrade an array of recalcitrant organic pollutants via highly reactive oxidants such as hydroxyl radical (·*OH*) and sulfate radical ($SO_{4}^{\;\cdot -}$) [14,15,16,17], among which, $SO_{4}^{\;\cdot -}$-based AOPs have recently attracted tremendous attention due to their strong oxidation ability and wide reaction pH range [18]. $SO_{4}^{\;\cdot -}$ can be generated from persulfate anion ($S_{2}O_{8}^{2-}$), as reported in many successful applications of the peroxydisulfate (PDS)-based oxidation process once activated by light, metal ion, or heat [15,19,20]. Then, in principle, persulfates could be a suitable candidate to be integrated with MD, where the temperature of the feed solution, usually kept at 40–100 °C, could serve as an existing activator for the generation of $SO_{4}^{\;\cdot -}$ [15,18]. The degradation of surfactants using AOPs, especially to mitigate membrane wetting in the MD process, has rarely been investigated.

We thus hypothesize that the wetting issue caused by trace surfactant for hydrophobic coupons could be mitigated given surfactant molecules were attacked and degraded by heat-activated radicals (more precisely, the activated PDS) in a hybrid MD-AOP. We further assume that the different wetting behavior of membrane coupons in varying operating conditions for such a hybrid process would confirm the occurrence and efficacy of the reaction proposed above. Previous findings display the potential benefit of integrating persulfate towards membrane fouling alleviation, where foulant compounds could be oxidized [16,21], in line with the validation via the membrane characterization. However, wetting dynamics in the MD process, given the AOP integration, are not easily detected on the basis of membrane coupon [22]. So far, the most commonly described method for the wetting phenomenon of such a hydrophobic membrane refers to monitoring the variation of vapor flux and distillate electrical conductivity, but it was not sensitive enough to reflect varying wetting extents in time [10,23,24]. Alternatively, impedance-based membrane monitoring is recommended for capturing the wetting by trace surfactant (e.g., 10 ppm Triton X-100), since it is capable of detecting the subtle variation that occurs at the membrane during the propagation of feed solution/air interface toward the distillate [25,26].

Therefore, this study aimed to testify the wetting mitigation hypothesis in PDS-assisted MD in dealing with surfactant containing salty feed. The effect of SDS concentration (i.e., 0, 0.5, and 0.8 mM), PDS concentration (i.e., 0, 1, 3 and 5 mM) and feed temperature (i.e., 55 or 65 °C) on MD performance was investigated and discussed. In addition, membrane characterizations (i.e., the impedance-based technique) were applied to monitor the membrane wetting to varying extents (particularly toward that under the anti-wetting scenario). The outcome of this study would be useful in developing potential wetting mitigation techniques and further exploring its efficacy in wider aspects of the MD process.

## 2. Materials and Methods

### 2.1. Materials

Two types of flat-sheet membranes, namely polyvinylidene fluoride (PVDF) (GVHP, Merck-Millipore, nominal pore diameter of 0.22 μm, thickness of 125 μm, and porosity of 75%) and polytetrafluoroethylene (PTFE) (GC-HPTFE, Guochu Technology, nominal pore diameter of 0.22 μm, thickness of 200 μm, and porosity of 80–90%) were used. Sodium dodecyl sulphate (SDS; anionic; MW = 288.4 g/mol), sodium peroxydisulfate (PDS), and sodium chloride (NaCl) were all reagent type (Aladdin Biochemical Technology Co., Ltd., Shanghai, China) and used as received without further purification. Deionized (DI) water (UPR-ΙΙ-10TN, Ulupure Technology Co., Ltd., Chengdu, China) was used for all experiments.

### 2.2. Direct Contact Membrane Distillation Set-Up

**Figure 1:**
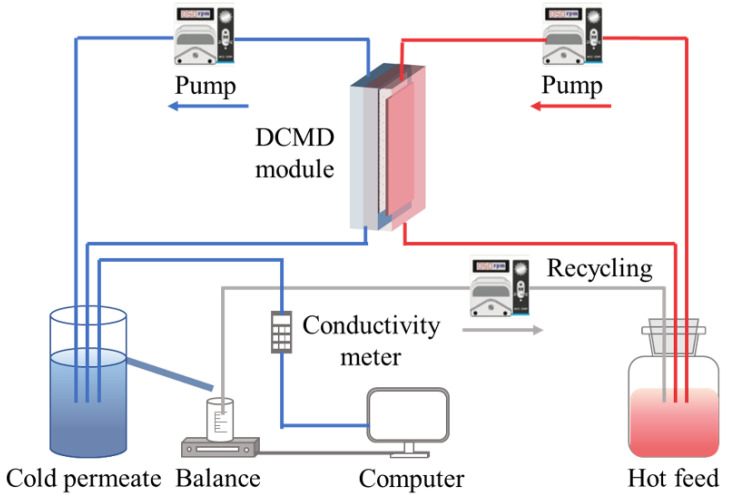
Schematic of the DCMD setup.

A direct contact membrane distillation (DCMD) cell (effective area of 38 cm^2^, 10.6 cm × 3.6 cm, and both the feed and permeate sides were the height of 2 mm) was used in MD experiments to evaluate membrane performance (Figure 1). The temperature of the feed (65 or 55 °C) and the distillate (15 °C) were maintained via the heater and chiller (Yiheng, China), respectively. The feed and distillate streams were circulated at cross-flow rates of 500 and 200 mL/min by two peristaltic pumps (Chuangrui, China). Initially, the MD system was operated using feed of only 5 g/L NaCl until the flux was stabilized after at least 4 h, and then, the SDS and/or PDS to be investigated was added to the feed tank. A peristaltic pump (BT100FJ, Chuangrui, China) was used to maintain the feed concentration approximately at a constant by recycling 30 mL from the overflow tank to the feed tank. The variation of permeate weight and its electrical conductivity was monitored on-line using a digital balance (Mettler-Toledo, Zurich, Switzerland) and conductivity meter (Hach, Loveland, CO, USA) to calculate the vapor flux (J, kg/m^2^/h) and salt rejection (R, %), respectively, according to Equations (1) and (2), respectively.

(1) $$J=\frac{m}{A\Delta T}$$

with m referring to the accumulated weight of permeate (kg) during the time interval for data record (denoted as ΔT, h), and A referring to the effective membrane surface area (m^2^).

(2) $$R\left(\%\right)=\left(1-\frac{C_{p}}{C_{f}}\right)\times 100\%$$

where C_f_ and C_p_ are conductivity (μS/cm) for the feed and the permeate, respectively.

### 2.3. Characterization

The membrane plain surface and cross-section morphologies were characterized using a scanning electron microscope (SEM, Hitachi, Tokyo, Japan). The surface wettability was characterized via the static contact angles at least 5 times using a goniometer (Shengding, China), which also afforded the determination of the liquid surface tensions [27]. Zeta potential of the membrane was evaluated using an electrokinetic analyzer (Anton Paar, Graz, Austria). Information regarding the functional groups on the membrane interface was studied using Fourier transform infrared spectroscopy (FTIR) (Shimadzu, Kyoto, Japan).

In addition, the membrane was also evaluated based on impedance test using a potentiostat (Admira, Squidstat plus, USA) with two electrodes (Haber–Luggin capillary, Ag/AgCl electrode immersed in 3.5 M KCl) on target coupons (effective area of 2.0 cm^2^), at a frequency from 10^−1^ Hz to 10^6^ Hz with a sinusoidal voltage of 100 mV at open circuit potential. Electrolyte solution of 1 M NaCl with a volume of 200 mL was used for this off-line analysis. EIS is a non-destructive technique in which a small-amplitude sinusoidal potential *E*(*t*) = *E*_0_sin(ωt) is applied to a system over a range of frequencies. The current response can be expressed as [28,29,30,31]:

(3) $$I\left(t\right)=I_{0}\sin\left(\omega t-\theta \right)$$

where $I_{0}$ and *θ* are the current amplitude and phase difference, respectively.

The impedance is given as below:

(4) $$Z=\frac{E\left(t\right)}{I\left(t\right)}=Z_{0}\left(\cos\omega +\sin\omega \right)=Z\prime -jZ\prime \prime$$

where *Z*′ and *Z*″ represent the real and imaginary impedance, respectively, and *j* is defined by J^2^ = −1.

## 3. Results and Discussion

### 3.1. Material Characterization

**Figure 2:**
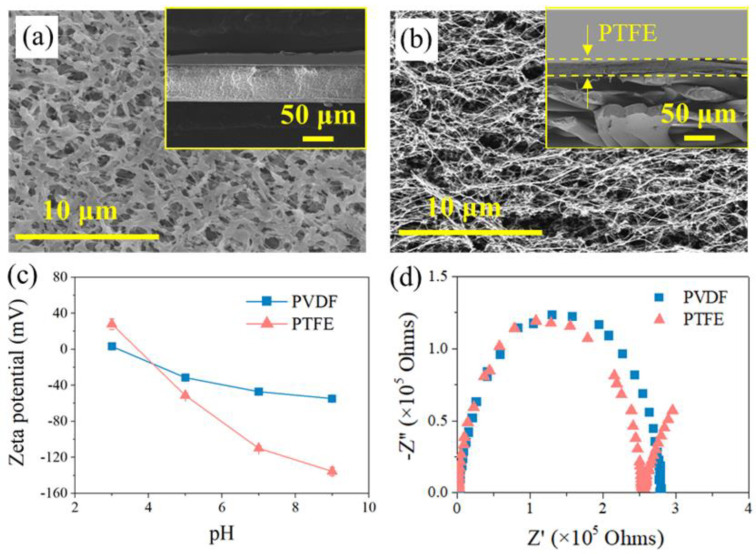
SEM images for membrane morphology of (**a**) PVDF and (**b**) PTFE, with the image for the membrane cross-section inserted. (**c**) Zeta potential of the pristine membranes at different pH values. (**d**) EIS data of the pristine membranes in electrolyte (1 M NaCl solution).

**Figure A1:**
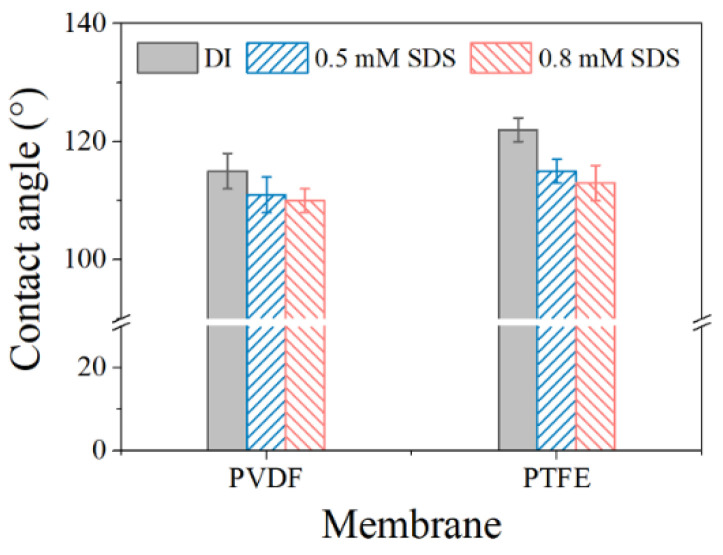
Contact angles of DI water, 0.5 mM SDS, and 0.8 mM SDS for the PVDF and PTFE membranes.

Figure 2a,b show that the PTFE membrane exhibited a spider-web-like, microporous structure compared with the irregularly porous network of the PVDF membrane, though both membranes have a similar nominal pore size of ca. 0.2 μm (data from the manufacturer). Regarding their cross-section structure, PTFE appeared to be two-layer structured, with a PP support layer and PTFE functional layer (~16 μm), while the PVDF membrane seemed homogenous, and the thickness of the PVDF film (ca.125 μm) was much smaller than its counterpart (the thickness of PTFE is 200 μm), shown in Figure 2b. At the herein investigated pH range (6~7), both membranes exhibited negative charge, with PTFE possessing more negativity (Figure 2c). In terms of the EIS analysis (Figure 2d), similar radii of the impedance curve for the two membranes were found, indicating a close hydrophobicity between the two investigated coupons. Indeed, the in-air contact angles (CAs) of the PVDF and PTFE membranes were quite comparable (115 ± 2° and 120 ± 3°, respectively), and the CA value of each membrane slightly decreased with lower surface energy liquids (50 and 45 mN/m for 0.5 mM and 0.8 mM SDS, respectively, in respect with ca. 72 mN/m for DI water) (Figure A1). Finally, the observed small arc for PTFE may be due to the membrane’s double-layered structure, consistent with the cross-section morphology of PTFE membrane. With such a different pair of two membrane coupons, the MD experiments were performed, and the results are illustrated in the following sections.

### 3.2. Effect of SDS Concentration on MD Performance

**Figure 3:**
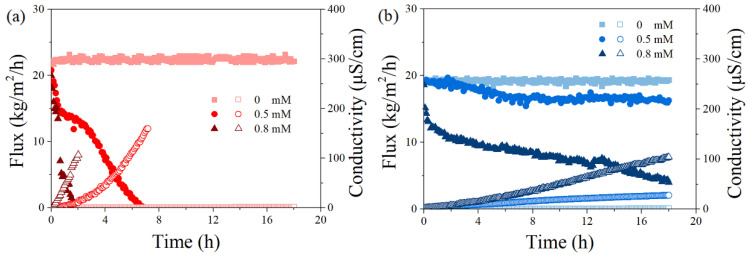
Effect of SDS concentrations (0, 0.5, and 0.8 mM) on permeate flux and permeate conductivity versus time for PVDF (**a**) and PTFE (**b**) for a feed with 5 g/L NaCl at the temperature gradients (T_f_–T_p_) of 50 °C, T_f_ = 65 °C. The flow rates for feed and permeate were 500 and 200 mL/min, respectively. The herein salt rejection rate (>99%) was not significantly different from each case and thus is not shown.

In order to investigate the effect of surfactants on the PVDF and PTFE membranes’ performance, a series of DCMD experiments were carried out under different concentrations of SDS (0–0.8 mM) with the salt solution (5 g/L NaCl). The flux and permeate conductivity over time are shown in Figure 3.

In the absence of SDS, both membranes exhibited stable performance with constant vapor flux (22 and 19 kg/m^2^/h for PVDF and PTFE, respectively) and extremely low permeate conductivity, an indicator of great permeate quality. The superior permeability of PVDF could be attributed to its membrane structure, such as a smaller thickness (125 vs. 200 μm for PVDF and PTFE, respectively), which possibly led to a smaller mass transfer resistance. However, membrane wetting was found in the presence of SDS, and the thinner PVDF scarified its vapor flux more in each case. With a 0.5 mM SDS solution, the flux of PVDF was completely lost after 6 h, while the PTFE membrane showed a small flux decline up to 16%, with the final permeate conductivity being significantly lower (28 vs. 160 μS/cm for PTFE and PVDF, respectively). When the SDS concentration was increased to 0.8 mM, the PVDF membrane sharply lost its vapor permeability within 2 h, while the flux of the PTFE membrane gradually dropped to a final value of 4 kg/m^2^/h, with the performance of their permeate conductivity in line with the flux behavior (not further extended). Previous studies have confirmed experimentally and theoretically that the SDS monomers tend to distribute on the membrane surface due to hydrophobic–hydrophobic interaction between the non-polar tails of the SDS and the hydrophobic membrane, and the adsorption of SDS would finally turn the membrane hydrophilic, partially wetted [32,33,34,35,36]. The present data agreed with the membrane wetting phenomenon by surfactant adsorption and highlighted the different behavior of two membrane coupons. Finally, considering a better investigation of anti-wetting performance under the presence of PDS, a moderate wetting case was selected in order to confirm and investigate the proposed fouling control effect, i.e., SDS concentrations of 0.5 and 0.8 mM were further used for experiments via the PVDF and PTFE membrane, respectively.

### 3.3. Effect of PDS Concentration and Feed Temperature

**Figure 4:**
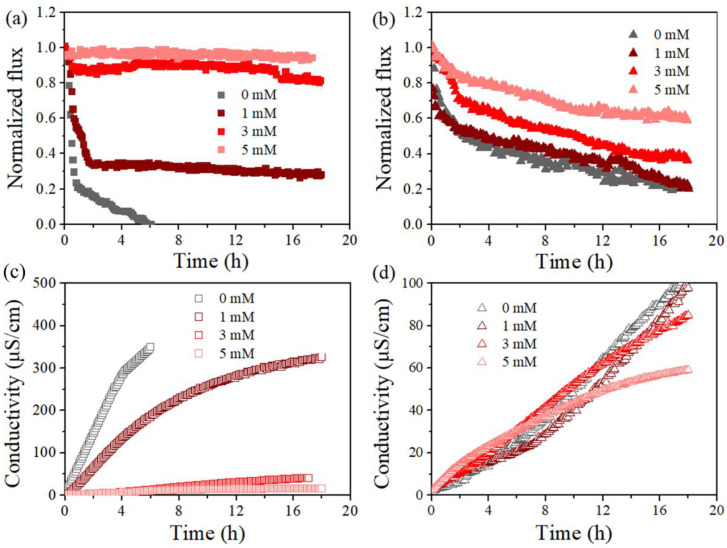
Effect of PDS concentrations (0, 1, 3, and 5 mM) on normalized, time-dependent permeability for PVDF (**a**,**c**) and PTFE (**b**,**d**) in the presence of 0.5 mM SDS (**a**,**c**) and 0.8 mM SDS (**b**,**d**), respectively. Normalization was on the basis of permeate flux for feed of 5 g/L NaCl in absence of SDS, which was 20.51 ± 1.81 kg/m^2^/h for PVDF, and 19.54 ± 1.52 kg/m^2^/h for PTFE at T_f_–T_p_ = 50 °C, respectively.

Figure 4 reports the variation of permeate flux and conductivity of the standalone and PDS-assisted DCMD dealing with SDS-contained saline solution. The anti-wetting effectiveness of the developed integrated process was found for each membrane coupon within the investigated range of PDS concentration. Indeed, the occurrence of flux decline and conductivity rise for the permeate were found in each experiment, but the extent appeared to be dependent on the PDS concentration. Taking PVDF membrane as an example, in the absence of PDS, the membrane vapor flux quickly dropped to 0 within 6 h (Figure 4a), and the initial flux drop could be attributed to a quick adsorption of SDS once dosed into the feed, which initiated partial wetting in some membrane areas. Then, in the presence of PDS, a slower flux decline was found in each condition, which gave final flux reduction as ca. 72%, 20%, and 5% for PDS concentration of 1, 3, and 5 mM, respectively. Accordingly, the conductivity of the permeate by the PDS-assisted DCMD system (6 h) ranged between 10 µS/cm (5 mM PDS) and 350 µS/cm (0 mM PDS). The fact that the rising rate of permeate conductivity slightly varied at different stages could be attributed to the evolution of the wetting degrees, from initial partial wetting at the membrane surface or pore level until a considerable number of membrane pores are finally filled with feed water. As for PTFE, flux reduction was also mitigated given PDS concentration at 0, 1, 3, and 5 mM, and the final flux dropped by 80%, 78%, 62%, and 40%, respectively. Thus, the addition of PDS in the MD feed clearly led to wetting mitigation. Note that the PVDF membrane behaved worse than PTFE when the PDS concentration was low, but it performed better at high concentrations. Such a finding may be attributed to a more sufficient reaction between PDS and SDS, i.e., the SDS concentration was lower for PVDF (0.5 mM) than its counterpart (0.8 mM for PTFE).

**Figure 5:**
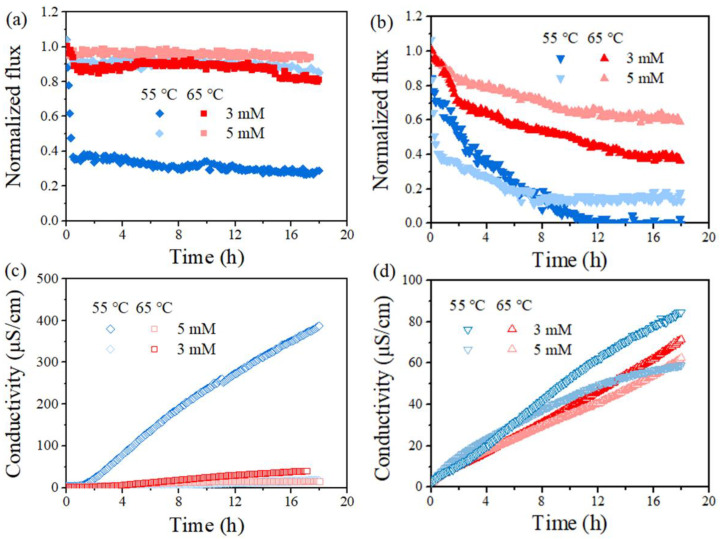
Effect of temperature (T_f_ = 65 °C and T_f_ = 55 °C) on normalized, time-dependent permeability for PVDF (**a**,**c**) and PTFE (**b**,**d**) in the presence of 0.5 mM SDS (**a**,**c**) and 0.8 mM SDS (**b**,**d**). Normalization was on the basis of permeate flux for feed without SDS yet of 5 g/L NaCl, which was 20.51 ± 1.81 for the PVDF and 19.54 ± 1.52 kg/m^2^/h for PTFE at T_f_–T_p_ = 50 °C, and 14.61 ± 1.26 kg/m^2^/h for PVDF and 14.52 ± 1.72 for PTFE at T_f_–T_p_ = 40 °C, respectively.

Figure 5 further compares the MD performance at a feed temperature of 55 °C and 65 °C, taking the cases with PDS concentration at 3 and 5 mM as examples. The herein observed impact of feed temperature was applicable to each case. Specifically, an increase in feed temperature led to a reduced final flux decline ratio from 72% to 20% and less final permeate conductivity (negligible compared with 387 μS/cm) for the PVDF membrane at a PDS concentration of 3 mM (Figure 5a,c). Such an enhancement of membrane performance was typically clearer for PVDF, probably due to the fact that the lower SDS amount (lower SDS concentration applied) was sufficiently oxidized compared to PTFE. Such an improvement of MD performance with feed temperature was also found at a higher PDS concentration, where the flux loss of the PVDF membrane was already very small and it is not further extended, since it is discussed above. In brief, at a higher feed temperature, both membranes exhibited wetting mitigation supported by smaller flux drop and a lower conductivity rise with the presence of PDS. Overall, these results demonstrated that the membrane wetting induced by SDS was modulated by PDS concentration and feed temperature.

### 3.4. Elucidating Membrane Wetting Extent

**Figure A2:**
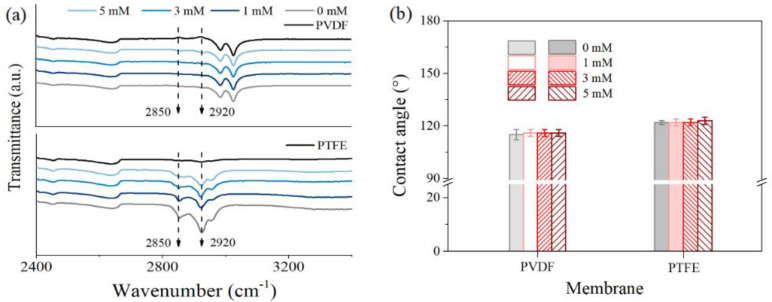
FTIR spectra of the pristine and used membranes (**a**), water contact angle for the testing in MD with different concentrations of PDS solution feeds according to Figure 4**b**.

Since the above fouling/wetting phenomenon in Figure 4 and Figure 5 was hypothesized to be the consequence of SDS attachment onto the membrane matrix under their hydrophobic–hydrophobic affinity, FTIR analysis was performed to confirm the existence of SDS on the used membranes (Figure A2) with respect to the pristine membranes. Note the information given by SDS moiety includes two peaks around 2926 cm^−1^ and 2851 cm^−1^, which were attributed to CH_2_ symmetric and asymmetric stretching, respectively [34,37]. The results indicated that the featured peak for SDS was only slightly observable on the PTFE membrane dealing with feed containing a relatively higher SDS quantity (null for the PVDF membrane dealing with feed of 0.5 mM SDS). Neither CA of both used membranes revealed clear variation for surface wettability after being exposed under the present suite of experiments (Figure A1 and Figure A2). We thus characterized the membrane fouling/wetting phenomenon with EIS, a technique sensitive enough to elucidate the subtle variation of interface properties.

**Figure 6:**
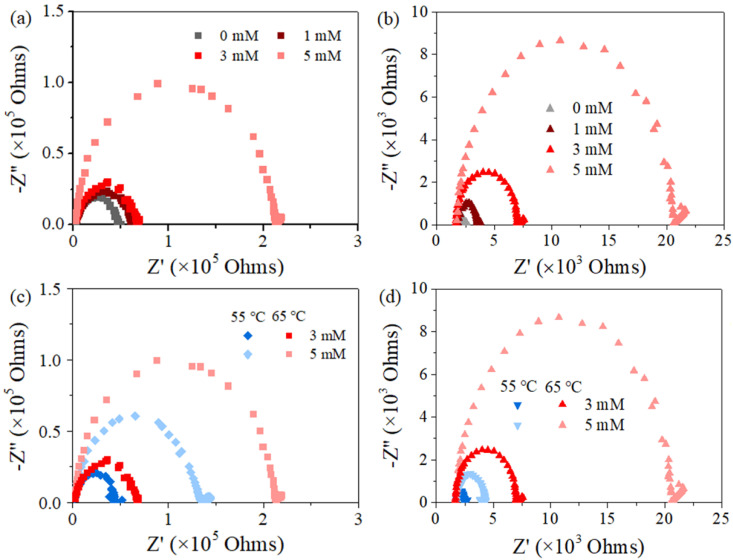
Nyquist plots of EIS data for fouled membrane of PVDF (**a**,**c**) and PTFE (**b**,**d**). Feed with different PDS concentrations (**a**,**b**) and temperatures (**c**,**d**) according to Figure 4 and Figure 5, respectively.

Nyquist plots present the correlation between the real component and the imaginary component of impedance at each frequency (Figure 6). All the spectrums shifted to the left and became smaller with declining PDS concentration from 5 mM to 0 mM (Figure 6a,b) and with declining feed temperature from 65 °C to 55 °C (Figure 6c,d). Moreover, the scale of impedance for PTFE was ca. 2 orders of magnitude smaller than PVDF. The above electrochemical information exhibits the same order of sequence with that for MD performance indicated by flux and conductivity variation in this study. In addition, the spectra for these used membranes were systematically smaller than their corresponding data for the pristine one (Figure 2).

**Figure A3:**
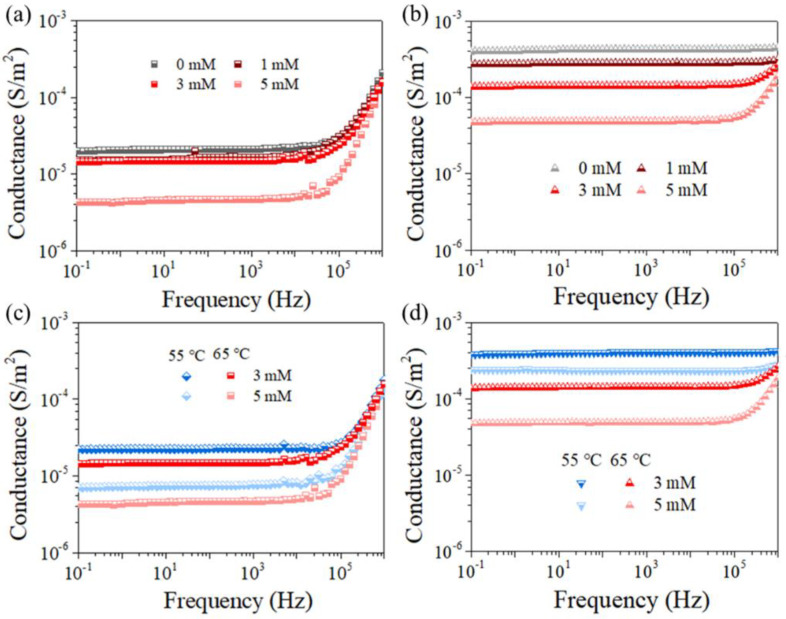
Conductance of PVDF (**a**,**c**) and PTFE (**b**,**d**) membrane versus MD duration for feeds with different PDS concentrations (**a**,**b**) and temperatures (**c**,**d**) according to Figure 4 and Figure 5, respectively.

**Figure A4:**
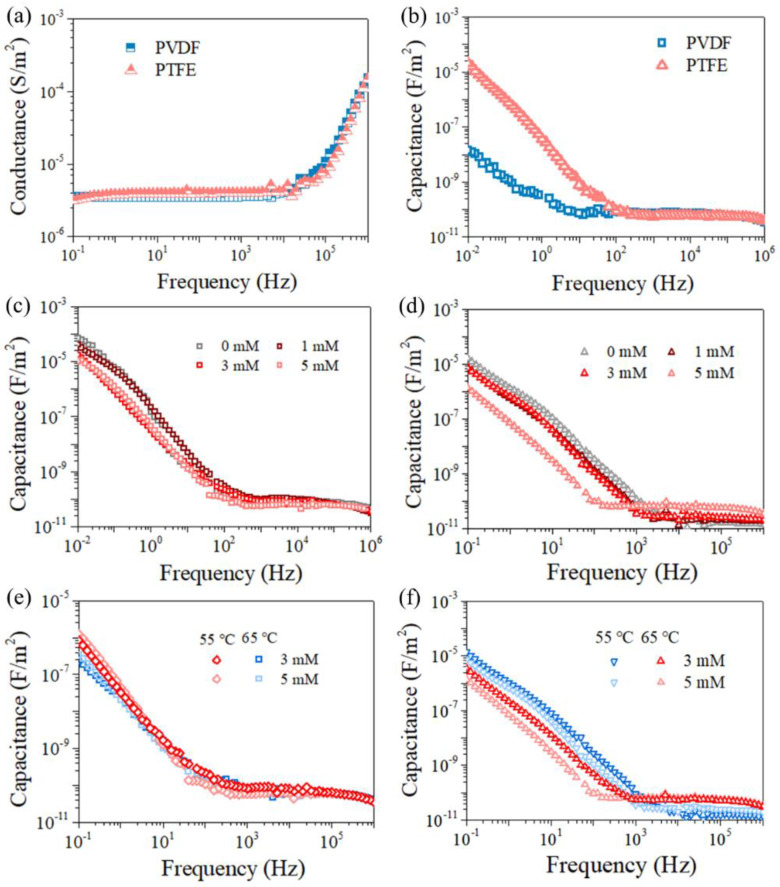
Conductance (**a**) and capacitance (**b**) of the pristine membranes in electrolyte (1 M NaCl solution), respectively. Capacitance of PVDF (**c**,**e**) and PTFE (**d**,**f**) membrane versus MD duration for feeds with different PDS concentrations (**c**,**d**) and temperatures (**e**,**f**) according to Figure 4 and Figure 5, respectively.

The physical meaning of the membrane impedance for the hydrophobic film largely correlates with its hydrophobicity, since the unique, stagnant air gap exists to construct a solid–liquid–gas triple phase in MD. Interestingly, the presence of an air gap explains such a high value of real and the imaginary impedance that is several orders of magnitudes greater than water-permeable and ion-permeable membranes [25,26,38,39,40,41,42]. Consequently, one could associate the membrane wetting behavior with the sensitive data obtained from EIS, and the shrank spectrum agreed with the occurrence of membrane wetting. Previous studies have argued that when the membrane was exposed to a lower-surface-tension liquid (e.g., SDS solution), gradual pore wetting could occur with decreased distance between the two liquid−air interfaces or propagated feed solution−air interface toward the distillate, until the final penetration of liquid into the membrane pore/matrix [25,26,41]. Then, the overall increased conductivity of the membrane in agreement with the loss of salt rejection can be expected, which is exactly the phenomenon shown in Figure A3, referring to the variation of conductance-frequency for different membrane coupons investigated (Figure A4). On the basis of the results above, we concluded by correlating those changes in EIS signals with anti-wetting performance in PDS-assisted MD systems dealing with surfactant contained feed.

### 3.5. Mitigation of Membrane Fouling by Heat-Activated PDS

The reduction in permeate flux reduction during the standalone DCMD operation could be attributed to membrane fouling/wetting. The amphiphilic surfactant could attach to the membrane surface via hydrophobic–hydrophobic interaction, which could result in the formation of a dense fouling layer on membrane surface. The fouling layer formed on the MD membrane limits the active area for the transport of water vapors; consequently, this can reduce permeate flux [34,37].

The much slower flux reduction for PDS-assisted DCMD can be attributed to the degradation of SDS achieved by heat-activated PDS. This could be attributed to the substantial chemical transformation of SDS, which results in of the loss of the amphiphilic property. This result agrees with previous studies in electrolysis at the BDD anode, where the release of the sulfate moiety from SDS was found [22,43,44].

Previous reports confirmed that heat served as a PDS activator, leading to the peroxide bond (O–O) to break in PDS and sulphate radicals ($SO_{4}^{\;\cdot -}$, E_0_ = 2.5–3.1 V) and/or hydroxyl (·OH, E_0_ = 2.8 V) radicals to be generated (Equations (5)–(7)) [16,20]. Then, we assume that the generated radicals could attack SDS molecules (with the investigated pH of the feed of 6 ± 1 falling into the range for the favored condition for PDS activation) [15]. Future work is warranted to further identify the attack or degradation pathway of the pollutants under the scenario of PDS-mediated oxidation. Meanwhile, further work is required to investigate the chemical kinetics between SDS and PDS. We believe the radicals formed in the MD system given the activation of PDS by feed heat could provide insight in membrane fouling/wetting control and/or target degradation in dealing with concentrated wastewater.

(5) $$S_{2}O_{8}^{2-}+heat\to 2SO_{4}^{\;\cdot -}$$

(6) $$SO_{4}^{\;\cdot -}+H_{2}O\to SO_{4}^{2-}+\cdot OH+H^{+}\;(pH<7)$$

(7) $$SO_{4}^{\;\cdot -}+OH^{-}\to SO_{4}^{2-}+\cdot OH\;(pH>7)$$

## 4. Conclusions

This work demonstrated that the PDS-assisted MD successfully alleviated wetting for the PVDF and PTFE membrane in desalinating low-surface-tension stream. Both the PDS concentration and feed temperature are important factors in such a hybrid system, where the heat activation of PDS was particularly specified. Two commercial membranes (PVDF and PTFE) were employed to operate with surfactant containing salty feed (SDS concentration up to 0.8 mM), which caused the wetting phenomenon for both membranes. The presence of PDS in the feed (concentration at 3 and 5 mM) obviously protected the membrane from significant wetting. Furthermore, superior performances were also achieved with the increase in the feed temperature. Interestingly, for varying membrane wetting scenarios, a wetting mitigation strategy based on the hybrid process was always found to be successful in reducing flux decline and conductivity rising, which was also well dictated by impedance-based monitoring on the membranes used. The proposed analytical approach via EIS seems sensitive enough to elucidate the phenomenon. The results suggest that PDS-assisted MD could probably alleviate membrane fouling/wetting and realize the in-situ treatment of complex streams simultaneously via the MD process.

## Data Availability

Not applicable.
